# One-stage tracheostomy during surgery reduced early pulmonary infection and mechanical ventilation length in complete CSCI patients

**DOI:** 10.3389/fsurg.2022.1082428

**Published:** 2023-03-17

**Authors:** Lin Sun, Haoyu Feng, Jun Mei, Zhiqiang Wang, Chen Deng, Zhixin Qin, Junqiao Lv

**Affiliations:** ^1^ Department of Orthopedics, Shanxi Bethune Hospital, Shanxi Academy of Medical Sciences, Tongji Shanxi Hospital, Third Hospital of Shanxi Medical University, Taiyuan, China; ^2^ Tongji Hospital, Tongji Medical College, Huazhong University of Science and Technology, Wuhan, China

**Keywords:** cervical spinal cord injury, tracheostomy, surgery, pulmonary infection, mechanical ventilation, risk factors

## Abstract

**Objective:**

Complete cervical spinal cord injury (CSCI) is a devastating injury that usually requires surgical treatment. Tracheostomy is an important supportive therapy for these patients. To evaluate the effectiveness of early one-stage tracheostomy during surgery compared with necessary tracheostomy after surgery, and to identify clinical factors for one-stage tracheostomy during surgery in complete cervical spinal cord injury.

**Design:**

Data from 41 patients with complete CSCI treated with surgery were retrospectively analyzed.

**Participants and interventions:**

Ten patients (24.4%) underwent one-stage tracheostomy during surgery, thirteen (31.7%) underwent tracheostomy when necessary after surgery, and eighteen (43.9%) did not have a tracheostomy.

**Main results:**

One-stage tracheostomy during surgery significantly reduced the development of pneumonia at 7 days after tracheostomy (*p* = 0.025), increased the PaO_2_ (*p* < 0.05), and decreased the length of mechanical ventilation (*p* = 0.005), length of stay (LOS) in the intensive care unit (ICU) (*p* = 0.002), hospital LOS (*p* = 0.01) and hospitalization expenses compared with necessary tracheostomy after surgery (*p* = 0.037). A high neurological level of injury (NLI) (NLI C5 and above), a high PaCO_2_ in the blood gas analysis before tracheostomy, severe breathing difficulty, and excessive pulmonary secretions were the statistically significant factors for one-stage tracheostomy during surgery in the complete CSCI patients, but no independent clinical factor was found.

**Conclusions:**

In conclusion, one-stage tracheostomy during surgery reduced the number of early pulmonary infections and the length of mechanical ventilation, ICU LOS, hospital LOS and hospitalization expenses, and one-stage tracheostomy should be considered when managing complete CSCI patients by surgical treatment.

## GRAPHICAL ABSTRACT

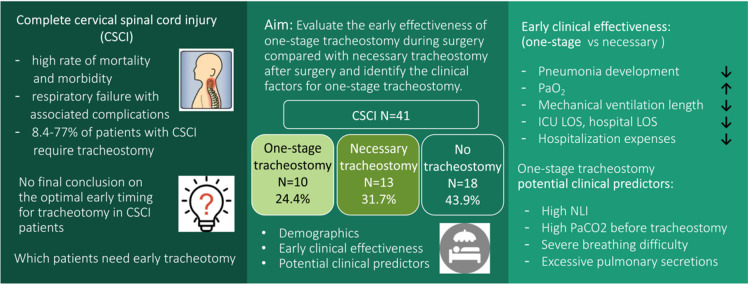

## Introduction

Complete cervical spinal cord injury (CSCI) is a devastating injury with a high rate of mortality and morbidity (1). The leading cause of early death after complete CSCI is respiratory failure with associated complications, and these are common due to the paralysis of the diaphragm and respiratory accessory muscles, as well as due to the excessive bronchial mucous production following complete CSCI (2, 3). Surgical stabilization and decompression is by far one of the best therapies for complete CSCI patients and is recommended (4–6).

Tracheostomy, as an important supportive therapy, is required in 8.4%–77% of patients with CSCI because it can improve their ventilatory function, decrease the risk of pulmonary complications, and save the patient's life (7, 8). As reported, the influencing factors for tracheostomy in CSCI patients include the patient's neurological severity of injury, as defined by the American Spinal Injury Association Impairment Scale (AIS), the patient's neurologic level of injury, hematoma-like changes on magnetic resonance imaging (MRI) scans, the injury seventy score (ISS), the volume of pulmonary secretions, smoking history, the associated injuries, and other factors (7, 9–12). Despite some risks and complications, the benefits of early tracheostomy, especially the reduction of the need for mechanical ventilation and the reduced intensive care unit (ICU) stay, in CSCI patients have been reported (11, 13, 14). However, there is no final conclusion on the optimal early timing for tracheotomy in CSCI patients, especially in complete CSCI patients who are probably the ones that are most in need of tracheotomy, and it is difficult to identify the patients who will require early tracheostomy while considering the expected benefits and risks.

The purpose of this retrospective study was to evaluate the early effectiveness of one-stage tracheostomy during surgery compared with necessary tracheostomy after surgery in complete CSCI patients having surgical treatment and to identify the clinical factors for one-stage tracheostomy during surgery in these patients.

## Methods

We conducted a retrospective single-center study in a university affiliated-tertiary referral hospital in China from January 2012 to June 2019, and this study was approved by the institutional review board. The inclusion criteria were as follows: patients aged 18 years or older; with cervical SCI between the levels of C3 and C7; with an AIS A grade; and who had surgical treatment. The exclusion criteria included tracheostomy before surgery, a significant craniocerebral or head injury, history of cerebral stroke, respiratory disease when the injury occurred, or death during hospitalization.

A total of 41 patients with complete CSCI treated with surgical treatment were included in the study. There were 18 patients in the no tracheostomy group, 13 patients in the necessary tracheostomy after surgery group, and 10 patients in the one-stage tracheostomy during surgery group. Also, we summarized the baseline characteristics of the patients such as age, gender. After surgery, the decision to perform a necessary tracheostomy was made by the spine surgeons together with intensive care unit (ICU) physicians. These decisions were based on the standards: if the individual patient had severe breathing difficulties, had blood gas disorders (some of the guidelines were a PaO_2_ less than 70 mmHg and a PaCO_2_ higher than 45 mmHg), and any factors which were associated with expected prolonged mechanical. Since December 2016, we performed the one-stage tracheostomy during surgery for the complete CSCI patients who had some risk factors before surgery but were not up to the standards of needing a necessary tracheostomy, such as difficulty breathing, excessive respiratory secretions, blood gas disorders, and a high level CSCI (C5 and above). The tracheostomies were performed after the incision for the cervical surgery was closed.

The tracheostomies were performed according to the standard surgical technique. Cervical spinal surgery was performed when the patient's condition was stable. Anterior cervical discectomy and fusion, anterior cervical corpectomy and fusion, or (and) posterior cervical laminectomy reduction and short segmental fixation using lateral mass screws were performed according to the cervical intervertebral disc injury or particular cervical facture and dislocation. Posterior unilateral open-door laminoplasty was performed in the patients who had spinal stenosis without fracture or dislocation.

First, the early clinical effectiveness of one-stage tracheostomy during surgery was compared with necessary tracheostomy after surgery in the complete CSCI patients having surgical treatment. The following parameters were recorded: the clinical pulmonary infection score (CPIS), it includes temperature, white blood cell count, tracheal secretion, oxygenation index, and chest x-ray, with a total score of 0 to 10. PaO_2_ according to a blood gas analysis, and PaCO_2_ according to a blood gas analysis before tracheostomy and at 1, 3, 5 and 7 days after the tracheostomy (15, 16). We also evaluated the length of time on mechanical ventilation, length of stay (LOS) in the intensive care unit (ICU) after tracheotomy, LOS of hospital stay, hospitalization expenses except for the cost of the cervical spinal surgery, tracheostomy closure at hospital discharge, and tracheostomy complications in both groups. The termination of mechanical ventilation was determined by the ICU doctors.

**Figure 1:**
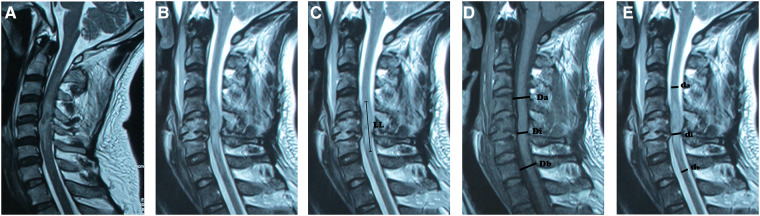
The level and severity of the cervical spinal cord injury on MRI. (**A**). The level of the cervical spine cord injury on MRI. Hematoma-like changes on MRI were defined as the presence of a hypointense core surrounded by a hyperintense rim in T2-weighted images, and if these findings were present, the level of hematoma-like changes served as the level of injury on MRI. (**B**). If hematoma-like changes were absent, the center of a wider-ranging hyperintensity in T2-weighted images served as the level of injury on MRI. Figure 1B also shows the most cephalic level of the hyperintensity in T2-weighted images. (**C**). Lesion length (LL) of the hyperintensity in T2-weighted images. (**D**). The maximum spinal canal compromise (MCC) in T1-weighted images. (**E**). The maximum spinal cord compression (MSCC) in T2-weighted images. **LL** represents the length of the lesion, which refers to the changing length of the spinal cord signal measured on the sagittal image. **MCC** was calculated based on the formula: (1- $\frac{\mathrm{Di}}{\left(\mathrm{Da}+\mathrm{DbY}2\right)}$), where **Di** is the diameter of the spinal canal at the level of spinal cord compression, **Da** is the normal diameter of the spinal canal above the spinal cord compression level, and **Db** is the normal diameter of the spinal canal below the spinal cord compression level. The **MSCC** was calculated based on the formula: (1 - $\frac{\mathrm{di}}{(\mathrm{da}+\mathrm{db})/2}$), where **di** represents the diameter of the spinal cord at the compression level, **da** represents the normal spinal cord diameter above the spinal cord compression level, and **db** represents the normal spinal cord diameter below the spinal cord compression level.

Then, the clinical factors potentially predicting the need for one-stage tracheostomy during surgery were studied. The data were extracted from the patient's medical records, and the data included the neurological level of injury (NLI), the overall injury severity expressed by the ISS (9, 17), the presence or absence of a vertebral fracture or dislocation at the level of cervical spinal injury, the level and severity of the cervical spine cord injury on MRI, the PaO_2_, PaCO_2_ and PaO_2_/FiO_2_ according to a blood gas analysis, difficulty breathing, excessive respiratory secretions (requiring hourly suction), history of smoking, and evidence of direct thoracic or abdominal trauma. In total, the clinicians identified nine factors (Table 1). The NLI was the most caudal segment that had normal motor and sensory functions on both sides of the body. Hematoma-like changes on MRI were defined as the presence of a hypointense core surrounded by a hyperintense rim in T2-weighted images, and if those were present, the level of hematoma-like changes was defined as the level of the cervical spine cord injury on MRI. If no hematoma-like changes were present, the center of a wider-ranging hyperintensity in T2-weighted images served as the level of injury on MRI (11). To evaluate the severity of injury on MRI, we examined the most cephalic level and the lesion length (LL) of the hyperintensity in T2-weighted images, and we also examined the maximum spinal canal compromise (MCC) and maximum spinal cord compression (MSCC) (18, 19). (Figure 1) Thoracic injury refers to frail chests, pneumothorax, or hemothorax, and abdominal injury refers to a solid organ injury higher than a grade of 2, combined with a visceral organ injury that required surgery (12).

**Table 1:** The clinical factors potentially predicting the need for one-stage tracheostomy during surgery in complete cervical spinal cord injury patients.

Variable	Type of date and definition	Type of measure
Neurological level of injury	C5 and above	1 = Yes.; 2 = No
Overall injury severity assessment Vertebral fracture and dislocation Magnetic resonance imaging (MRI) Blood gas analysis before tracheotomy Dyspnea Respiratory secretion Smoking Thoracic and abdominal trauma	Injury seventy score (ISS) Categorical Level of injury on MRI (C5 and above) Cephalic level of hyperintensity in T2 (C4 and above) Lesion length (LL) of hyperintensity in T2 Maximum spinal canal compression (MCC) Maximum spinal cord compression (MSCC) PaO_2_ (The lowest monitored in 24 h before tracheotomy) PaCO_2_ (The highest monitored in 24 h before tracheotomy) PaO_2_/FiO_2_ (The lowest monitored in 24 h before tracheotomy) Visible difficulty in breathing Significant phlegm (requirement for hourly suction) Categorical Categorical	Continuous 1 = Yes.; 2 = No 1 = Yes.; 2 = No 1 = Yes.; 2 = No Continuous Continuous Continuous Continuous Continuous Continuous 1 = Yes; 2 = No 1 = Yes; 2 = No 1 = Yes; 2 = No 1 = Yes; 2 = No

The continuous normally distributed variables are expressed as the mean ± standard deviation, the continuous nonnormally distributed data are expressed as the median and min-max range for skewed distributions, and the categorical variables are expressed as counting data and the rates. In the subgroup analysis, the early clinical effectiveness of tracheostomy was analyzed by using analysis of variance (ANOVA) followed by Dunnett's test for the data that was in accordance with the normal distribution and homogeneity of variance and by using a nonparametric rank sum test for the data that did not conform to normality. The effectiveness for one-stage tracheostomy during surgery compared with necessary tracheostomy after surgery was analyzed using the independent samples Student's t test for the normally distributed unpaired continuous data, the nonparametric rank sum test for the skewed data, and the chi-square test for the counting data and rates.

To identify the clinical factors for one-stage tracheostomy during surgery, univariate analyses were first carried out. ANOVA followed by Dunnett's test was used to compare the data that were in accordance with normality and homogeneity of variance, and a nonparametric rank sum test was used to compare the data that did not conform to normality. To further judge whether a factor was an independent influencing factor, a multivariate logistic regression analysis was conducted. The significance level was set at a *p* value less than 0.05. The statistical package SPSS version 22.0 (SPSS Inc., Chicago, IL, United States) was used for the analysis.

## Results

### Demographics

Forty-one patients with complete CSCI treated by surgical treatment were included in the analysis. The average age was 47.3 ± 13.8 years, and 86% were male. The overall median ISS was 25 (IQR: 25, 41), and the median anatomic level of the cervical spinal cord injury was 5 (IQR: 4, 7). The overall length of time from injury to cervical spinal surgery was 5.8 ± 3.24 days. The cervical spinal surgery of each patient at admission revealed that 30 of the 41 patients underwent anterior surgery, 6 underwent posterior surgery, and 5 underwent anterior surgery combined with posterior surgery.

Ten patients (24.4%) underwent one-stage tracheostomy during surgery, thirteen (31.7%) underwent tracheostomy when necessary after surgery (the length of time from cervical spinal surgery to tracheostomy was 6.5 ± 4.24 days), and eighteen (43.9%) did not have a tracheostomy. The demographic and clinical data for the one-stage tracheostomy during surgery, necessary tracheostomy after surgery, and no tracheostomy subgroups are summarized in Table 2.

**Table 2:** The demographic and clinical data for the one-stage tracheostomy during surgery, necessary tracheostomy after surgery, and no tracheostomy subgroups.

	One-stage tracheotomy	Postoperative tracheotomy	No tracheotomy	*p*
Age				
Mean ± SD (years)	48 ± 7.9	49 ± 17.1	46.8 ± 13.4	0.903
Sex				
Male	*n* = 8,80%	*n* = 12,92%	*n* = 14,78%	0.836
Female	*n* = 2,20%	*n* = 1,8%	*n* = 4,22%	
Spinal injury level				
C4	*n* = 3,30%	n = 6,46%	*n* = 1,5.5%	0.734
C5	*n* = 7,70%	n = 6,46%	*n* = 9,50%	
C6	*n* = 0	n = 1,8%	*n* = 7,39%	
C7	*n* = 0	n = 0	*n* = 1,5.5%	
Injury to operation time				
Mean ± SD (days)	5.8 ± 4.02	4.3 ± 3.02	6.9 ± 2.35	0.094
Surgical method				
Anterior	*n* = 5,50%	*n* = 11,84%	*n* = 14,78%	0.828
Posterior	*n* = 3,30%	*n* = 1,8%	*n* = 2,11%	
Anterior + posterior	*n* = 2,20%	*n* = 1,8%	*n* = 2,11%	

### Early effectiveness of one-stage tracheostomy during surgery compared with necessary tracheostomy after surgery for complete CSCI treated by surgical treatment

Before tracheostomy, the CPIS in the one-stage tracheostomy during surgery group was significantly lower than that in the necessary tracheostomy after surgery group. At 7 days after tracheostomy, the CPIS in the one-stage tracheostomy group was significantly decreased compared with that before tracheostomy (*p* = 0.025). However, the CPISs in the necessary tracheostomy after surgery group at 1, 3, 5 and 7 days after tracheostomy were no different compared with those before tracheostomy (*p* > 0.05). The CPISs were significantly lower in the one-stage tracheostomy during surgery group than those in the necessary tracheostomy after surgery group at 1, 3, 5 and 7 days after tracheostomy (Table 3).

**Table 3:** The clinical pulmonary infection score (CPIS) for the one-stage tracheostomy during surgery group and the necessary tracheostomy after surgery group before tracheostomy and at 1, 3, 5 and 7 days after tracheostomy.

	Before tracheostomy	1 day after tracheostomy	3 days after tracheostomy	5 days after tracheostomy	7 days after tracheostomy
One-stage tracheostomy during surgery	4.3 ± 1.1	4.3 ± 1.6	5 ± 1.1	4.7 ± 1.3	3 ± 1.1
Necessary tracheostomy after surgery	6.5 ± 1.2	6.3 ± 1.4	6.8 ± 1.2	6.3 ± 0.9	5.6 ± 1.0
t	4.588	3.379	3.822	3.663	5.931
*p*	<0.001	0.03	0.001	0.01	<0.001

Before tracheostomy, the PaO_2_ according to the blood gas analysis in the one-stage tracheostomy during surgery group was significantly higher than that in the necessary tracheostomy after surgery group. The PaO_2_ in both groups at 1, 3, 5 and 7 days after tracheostomy was significantly increased compared with that before tracheostomy (*p* < 0.05). The PaO_2_ was higher in the one-stage tracheostomy during surgery group than in the necessary tracheostomy after surgery group at 1, 3, 5 and 7 days after tracheostomy, but the difference was not statistically significant (Table 4).

**Table 4:** Pao_2_ according to the blood gas analysis for the one-stage tracheostomy during surgery group and the necessary tracheostomy after surgery group before tracheostomy and at 1, 3, 5 and 7 days after tracheostomy.

	Before tracheostomy	1 day after tracheostomy	3 days after tracheostomy	5 days after tracheostomy	7 days after tracheostomy
One-stage tracheostomy during surgery	75.0 ± 16.7	90.2 (70,244)	111.7 ± 32.8	109.3 ± 22.2	109.2 ± 18.4
Necessary tracheostomy after surgery	61.0 ± 9.3	98.1 (56.5,183)	105.3 ± 21.4	103.6 ± 22.2	96.9 ± 18.3
t	2.373		0.567	0.614	1.591
*p*	0.033	0.71	0.577	0.546	0.127

The PaCO_2_ before tracheostomy in the one-stage tracheostomy during surgery group was significantly lower than that in the necessary tracheostomy after surgery group. The PaCO_2_ in the one-stage tracheostomy group at 1, 3, 5 and 7 days after tracheostomy was not different from that before tracheostomy (*p* > 0.05). The PaCO_2_ in the necessary tracheostomy after surgery group at 1, 5 and 7 days after tracheostomy was significantly decreased compared with that before tracheostomy (*p* < 0.05), but there was no difference at 3 days after tracheostomy compared with that before tracheostomy (*p* = 0.156). The PaCO_2_ was lower in the one-stage tracheostomy during surgery group than in the necessary tracheostomy after surgery group at 1, 3, 5 and 7 days after tracheostomy, but the difference was not statistically significant at 3, 5 and 7 days after tracheostomy (Table 5).

**Table 5:** Paco_2_ according to the blood gas analysis for the one-stage tracheostomy during surgery group and the necessary tracheostomy after surgery group before tracheostomy and at 1, 3, 5 and 7 days after tracheostomy.

	Before tracheostomy	1 day after tracheostomy	3 days after tracheostomy	5 days after tracheostomy	7 days after tracheostomy
One-stage tracheostomy during surgery	37.8 ± 4.7	35 ± 12.7	36.3 ± 5.2	37 ± 4	36.9 ± 3.1
Necessary tracheostomy after surgery	48.5 ± 12.4	39.3 ± 4.7	42.2 ± 9	39.7 ± 4.6	39.7 ± 3.4
t	2.832	2.538	1.853	1.497	2.002
*p*	0.012	0.019	0.078	0.149	0.058

Patients who underwent one-stage tracheostomy during surgery had a shorter length of mechanical ventilation, ICU LOS, and hospital LOS than the patients who underwent tracheostomy after surgery when it was necessary. The hospitalization expenses except for the cervical spinal surgery cost were also lower in the one-stage tracheostomy during surgery group. Most of the patients had an unsuccessful tracheostomy closure at hospital discharge, but the rate of successful tracheostomy closure at hospital discharge was higher in the one-stage tracheostomy during surgery group than in the necessary tracheostomy after surgery group. The length of mechanical ventilation, ICU LOS, hospital LOS, hospitalization expenses and tracheostomy closure in both groups are presented in Table 6. There were no complications related to tracheostomy in either of the groups during the hospital stay.

**Table 6:** The length of mechanical ventilation, length of stay (LOS) in the intensive care unit (ICU), hospital LOS, hospitalization expenses except for the cervical spinal surgery cost and the rate of successful tracheostomy closure at hospital discharge for the one-stage tracheostomy during surgery and necessary tracheostomy after surgery subgroups.

	Length of mechanical ventilation (days)	ICU LOS (days)	Hospital LOS (days)	Hospitalization expenses except for surgery (CNY)	Rate of successful tracheostomy closure
One-stage tracheostomy during surgery	5.3 ± 1.62	12.6 ± 2.7	25.7 ± 3.2	146,379.1 ± 28,684.5	20%
Necessary tracheostomy after surgery	9 ± 3.9	16.5 ± 2.5	33.4 ± 8.8	186,710.4 ± 55,811.5	16%
t/X^2^	3.208	3.56	2.927	2.248	9.783
*p*	0.005	0.002	0.01	0.037	0.002

### Clinical factors for one-stage tracheostomy during surgery for complete CSCI treated by surgical treatment

The univariate analyses of the clinical factors for tracheostomy revealed that high NLI (NLI C5 and above), increased PaCO_2_ in the blood gas analysis before tracheostomy, severe difficulty breathing, and significant pulmonary secretions (requiring hourly suction) were important categorical discriminators of tracheostomy in CSCI patients. Compared with the patients who did not receive a tracheostomy, the patients who underwent one-stage tracheostomy during surgery had a significantly higher NLI (*p* = 0.013), a higher PaCO_2_ (*p* = 0.039), more severe difficulty breathing (*p* < 0.001), and more pulmonary secretions (*p* < 0.001). Moreover, the patients who underwent necessary tracheostomy after surgery had a significantly higher NLI (*p* = 0.026), a higher PaCO_2_ (*p* < 0.001), lower PaO_2_ (*p* < 0.001) and PaO2/FiO2 (*p* < 0.001), more severe difficulty breathing (*p* < 0.001), and more pulmonary secretions (*p* < 0.001) than the patients who did not receive a tracheostomy.

However, between the patients in the one-stage tracheostomy during surgery group and those in the necessary tracheostomy after surgery group, there was no significant difference in the NLI (*p* = 0.370), PaCO_2_ (*p* = 0.313), difficulty breathing (*p* = 0.846), or pulmonary secretions (*p* = 0.092). The patients' PaO_2_ (*p* = 0.021) and PaO2/FiO2 (*p* = 0.009) in the necessary tracheostomy after surgery group were significantly lower than those in the one-stage tracheostomy during surgery group.

The ISS, vertebral fracture or dislocation at the level of cervical spine injury, the level and severity of the cervical spine injury on MRI, history of smoking, and evidence of direct thoracic or abdominal trauma were not significantly different among the three groups. The clinical factors and radiological factors are presented in Table 7.

**Table 7:** Univariate analyses of the clinical factors for the one-stage tracheostomy during surgery, necessary tracheostomy after surgery and no tracheostomy subgroups.

Variable	N (41)	One-stage tracheostomy during surgery (*n*=10)	Necessary tracheostomy after surgery (*n*=13)	No tracheotomy (*n*=18)	*p*-Value
Neurological level of injury:					
C_5_ and above	32	10 (100%)	12 (92.3%)	10 (55.6%)	0.008
Under the C_5_	9	0 (0)	1 (7.7%)	8 (44.4%)	
Overall injury severity assessment (ISS)	41	28.40±4.50	27.31±4.73	26.67±4.02	0.538
Vertebral fracture and dislocation					
YES	30	7 (70%)	9 (69.2%)	14 (77.8%)	0.840
NO	11	3 (30%)	4 (30.8%)	4 (22.2%)	
Level of injury on MRI					
C_5_ and above	28	7 (70%)	10 (76.9%)	11 (61.1%)	0.641
Under the C_5_	13	3 (30%)	3 (23.1%)	7 (38.9%)	
Cephalic level of hyperintensity in T2					
C_4_ and above	32	7 (70%)	11 (84.6%)	14 (77.8%)	0.703
Under the C_4_	9	3 (30%)	2 (15.4%)	4 (22.2%)	
Lesion length (LL) of hyperintensity in T2	41	2.36±0.917	2.31±1.052	2.33±1.163	0.885
Maximum spinal canal compression	41	0.531±0.111	0.536±0.110	0.587±0.154	0.439
Maximum spinal cord compression	41	0.651±0.099	0.689±0.123	0.713±0.152	0.497
PaO_2_ (The lowest monitored in 24 h before tracheotomy)	41	75.01±16.74	61.038±9.29	77.85±14.66	0.006
PaCO_2_ (The highest monitored in 24 h before tracheotomy)	41	37.83±4.73	48.47±12.43	31.706±4.29	0.000
PaO_2_/FiO_2_ (The lowest monitored in 24 h before tracheotomy)	41	293.80±112.58	175.08±95.70	338.61±101.14	0.000
Dyspnea					
YES	18	8 (80%)	10 (76.9%)	0 (0)	0.000
NO	23	2 (20%)	3 (23.1%)	18 (100%)	
Massive respiratory secretion					
YES	17	6 (60%)	11 (84.6%)	0 (0)	0.000
NO	24	4 (40%)	2 (15.4%)	18 (100%)	
Smoking					
YES	17	2 (20%)	7 (53.8%)	8 (44.4%)	0.248
NO	24	8 (80%)	6 (46.2%)	10 (55.6%)	
Thoracic and abdominal trauma					
YES	15	3 (30%)	6 (46.2%)	6 (33.3%)	0.676
NO	26	7 (70%)	7 (53.8%)	12 (66.7%)	

The aforementioned items with significant differences were further analyzed using multivariate logistic regression. The results revealed that there was no independent clinical factor for one-stage tracheostomy during surgery in the complete CSCI patients treated by surgery who are in the one-stage tracheostomy during surgery, necessary tracheostomy after surgery and no tracheostomy subgroups (*p* > 0.05).

## Discussion

A complete CSCI is a low-incidence but devastating event (1). Patients with a complete CSCI are more likely to require a tracheostomy than those with an incomplete CSCI (10–12, 20). The aims of the surgical treatment for patients with complete CSCI include the prevention of secondary SCIs, cervical spinal stabilization, early and better training of respiratory muscles, and improved expectoration (2, 3). As reported, it is not preferable to treat patients with complete SCI within the early (less than 24 h from injury) surgical timeframe (4, 6). The patients in our study received cervical spinal surgery 5.8 ± 3.24 days after the injury, which was when they were in a stable condition. In this study, to evaluate the early effectiveness and clinical factors of one-stage tracheostomy during surgery for complete CSCI patients treated with surgery, we excluded the complete CSCI patients who underwent necessary tracheostomy before surgery because these patients could not wait until the cervical spine surgery before having a tracheostomy. Because this study's complete CSCI patients' causes of death were not associated with tracheostomy, except for respiratory complications, we also excluded the complete CSCI patients who died during hospitalization (2, 7, 11).

The benefits of early tracheostomy for CSCI patients have been reported in some studies (8, 9, 11, 14, 21). However, tracheostomy is an invasive procedure with intrinsic risks (21, 22). We hold the opinion that tracheostomy before surgery may increase the risk of incisional infections in CSCI patients treated with anterior cervical surgery because the anterior cervical surgery incision is very close to the tracheostomy incision. Therefore, in the complete CSCI patients who had some clinical factors, we performed a one-stage tracheostomy, which was also an early tracheostomy, during their cervical surgery. Moreover, because these procedures were performed under general anesthesia and with the patients intubated, one-stage tracheostomy during surgery could also decrease some of the intrinsic risks of tracheostomy.

In this study, one-stage tracheostomy during surgery significantly reduced the pulmonary infection at 7 days after tracheostomy and decreased the length of mechanical ventilation, ICU LOS, hospital LOS and hospitalization expenses except for the cervical spinal surgery cost, as compared with necessary tracheostomy after surgery in the patients with complete CSCI treated with surgical treatment. As some authors have reported, the benefits of early tracheostomy in CSCI patients include improved respiratory expectoration, improved ventilator support, a shorter length of mechanical ventilation, prevention of pulmonary complications, and decreased ICU and hospital LOS (8, 11, 13, 14, 21, 23). However, other studies have indicated that early tracheostomy did not prevent respiratory complications and decreased the length of mechanical ventilator support (12, 22). In these studies, the controversial effect of early tracheostomy may result from the different constituent ratios of the CSCI patients evaluated, especially with regard to the different ratios of complete CSCI patients, because the patients with incomplete CSCI were less likely to require a tracheostomy (7, 23).

The development of pulmonary infections is common when there is paralysis of the diaphragm and respiratory accessory muscles, as well as excessive bronchial mucous production in patients with complete CSCI, and these are the most common etiologies for the morbidity and mortality in these patients (2, 3, 23). Patients who underwent necessary tracheostomy after surgery usually had more severe pulmonary infections as well as a lower PaO_2_ and a higher PaCO_2_ according to the blood gas analysis during the tracheostomy, as compared with patients who underwent one-stage tracheostomy during surgery. Sequentially, these patients may need longer mechanical ventilation and were more likely to fail extubation after tracheostomy. The length of mechanical ventilation is one of the important factors associated with the ICU LOS, hospitalization expenses, and ventilator-associated pneumonia mortality (8, 20). The reduction in the number of early pulmonary infections after one-stage tracheostomy during surgery was vitally helpful to decrease the length of mechanical ventilation. However, because of their pulmonary care needs, in our study, most patients did not have a successful tracheostomy closure at hospital discharge.

The risk factors for tracheostomy in patients with CSCI may include the neurological severity of the injury, the NLI, the ISS, the Glasgow Coma Scale (GCS) score, vertebral fracture and dislocation, hematoma-like changes on MRI, the variables for forced vital capacity, the volume of pulmonary secretions, the gas exchange, respiratory complications, smoking, thoracic and abdominal trauma, sex, and age, as reported in different studies (7–14, 19, 20, 23, 24). For the complete CSCI patients, a high NLI (NLI C5 and above) was a factor for one-stage tracheostomy during surgery, although it was not an independent clinical factor. Because of the chest wall and abdominal muscle paralysis in complete CSCI patients, the diaphragm is mainly responsible for the patient's respiratory function and is primarily innervated by C4 and partially innervated by C3 and C5. Consequently, many studies have indicated that an upper CSCI is an influential factor for tracheotomy, even early tracheotomy, for complete CSCI patients (7, 11–13, 23, 25).

The pulmonary ventilation of complete CSCI patients was also important to predict the need for one-stage tracheostomy during surgery. In spite of some shortcomings, the variable forced vital capacity is able to reflect the pulmonary ventilation and is considered indispensable to predict the need for tracheostomy in patients with CSCI during the acute stage (9, 19, 20). However, we have not conventionally measured the forced vital capacity in our hospital because no simple spirometer has been available. Visibly, we determined the difficulty in breathing, and used it as an indicator to estimate the capacity of the pulmonary ventilation. Pulmonary secretions are another crucial factor that affects the pulmonary ventilation. PaCO_2_ in the blood gas analysis is also a simple and convenient pulmonary ventilation index. Our results indicated that decreased pulmonary ventilation, including a high PaCO_2_ in the blood gas analysis, severe difficulty breathing, and significant pulmonary secretions, combined with a high NLI (NLI C5 and above), were the factors associated with one-stage tracheostomy during surgery for complete CSCI patients treated surgically.

This study has several limitations. First, it was a retrospective study that reflected the experience at a specialized spinal center in a university affiliated-tertiary referral hospital in China. Second, although complete CSCI is a low-incidence event, the sample size of the study is relatively small. Third, a longer follow-up time is needed to assess the effects of one-stage tracheostomy during surgery in complete CSCI patients with surgical treatment.

In conclusion, one-stage tracheostomy during surgery reduced the number of early pulmonary infections, the length of mechanical ventilation, the ICU LOS, the hospital LOS and hospitalization expenses when compared with necessary tracheostomy after surgery in complete CSCI patients treated surgically. Therefore, one-stage tracheostomy should be considered in complete CSCI patients with a high NLI, a high PaCO_2_ in the blood gas analysis, severe difficulty breathing, and significant pulmonary secretions.

## Data Availability

The raw data supporting the conclusions of this article will be made available by the authors, without undue reservation.
